# A Partial Anomalous Pulmonary Venous Connection in a Severely Symptomatic Patient, Is Surgery Always Recommended?

**DOI:** 10.7759/cureus.2962

**Published:** 2018-07-11

**Authors:** Charl Khalil, Wassim Mosleh, Amira Ibrahim, Herbert Young, John Corbelli

**Affiliations:** 1 Division of Medicine, State University of New York at Buffalo, Buffalo, USA; 2 Division of Cardiology, VA Western New York Health Care System, Buffalo, USA

**Keywords:** papvc, congenital, anomaly, surgery, intervention

## Abstract

Partial anomalous pulmonary venous connection (PAPVC) is a rare cardiac anomaly occurring when a pulmonary vein drains into the right atrium, coronary sinus or a systemic vein creating a left-to-right shunt. Symptoms develop from right-sided fluid overload and pulmonary vascular disease. We report a rare case of a severely symptomatic patient with an incidentally discovered PAPVC in the setting of underlying severe pulmonary hypertension from multifactorial severe restrictive lung disease. Despite his worsening symptoms, a multi-disciplinary meeting decided against surgical intervention. Nine months after the decision was made, the patient showed no signs or symptoms of clinical deterioration. Prior studies recommend surgery for PAPVCs with evidence of right ventricular dilation, mild-to-moderate tricuspid regurgitation, or early stages of pulmonary vascular disease. However, our case demonstrates how decision making should consider the shunt’s contribution to the overall clinical picture and underlying comorbidities. If a decision is made to defer surgical intervention, strict follow up and repeat re-evaluations for possible risk re-stratification and surgery reconsideration are warranted.

## Introduction

Partial anomalous pulmonary venous connection (PAPVC) is a rare cardiac anomaly usually incidentally identified on imaging [1-3]. It occurs when one or more of the pulmonary veins drain into the right atrium, coronary sinus or a systemic vein. Its prevalence is approximately 0.4%-0.7% in the general population, with 10% of cases being left-sided [1]. PAPVC creates a left-to-right shunt responsible for the associated symptomatology [2]. Symptoms would develop secondary to right-sided fluid overload, pulmonary vascular disease, and worsening right-sided heart failure [1, 3-4]. In isolated left-sided PAPVC with an intact atrial septum, the patient would usually be asymptomatic in childhood until it is incidentally discovered or symptoms are induced in adulthood [5]. Patients could present with dyspnea, fatigue, palpitations, chest pain, or peripheral edema [5]. Classically, surgical repair of PAPVC aims at draining the anomalous vein to the left atrium [6-7]. Surgical correction outcomes are favorable with low morbidity [8]. Cases managed by a novel robotically assisted minimally invasive technique has been also reported [9].

## Case presentation

**Figure 1:**
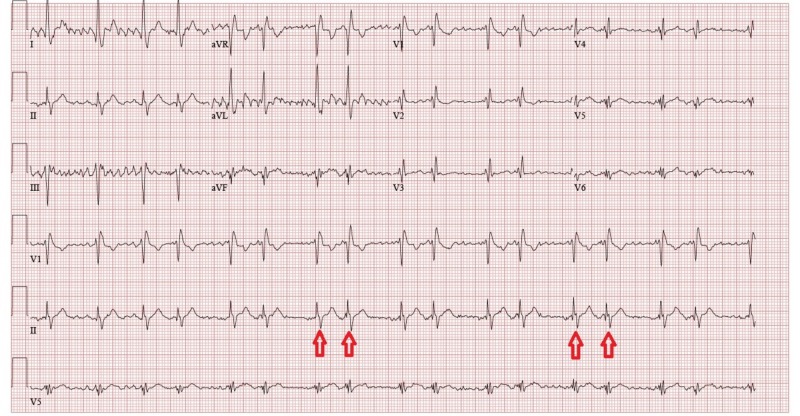
EKG EKG showing new onset atrial bigeminy (red arrows).

**Figure 2:**
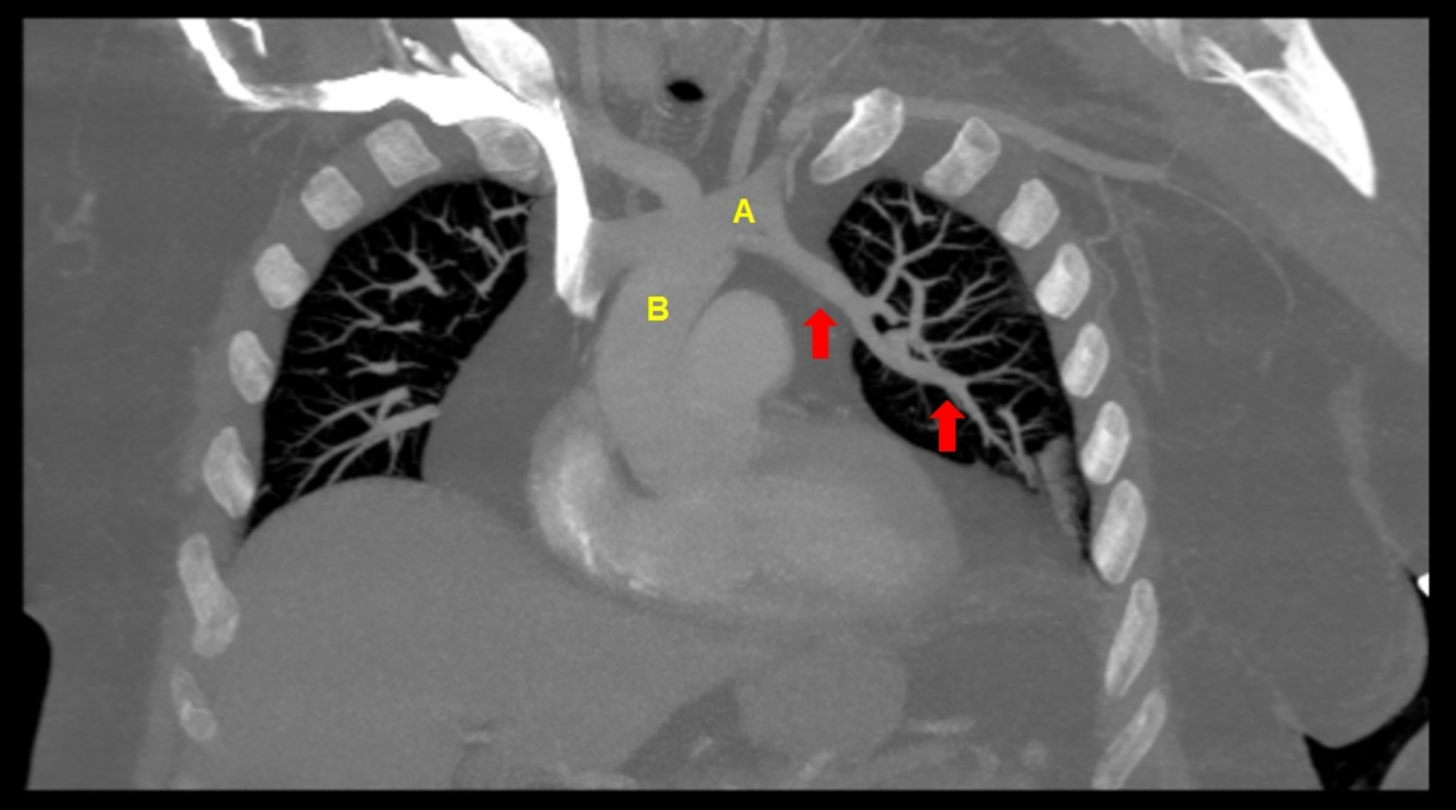
CT-angiogram coronal plan showing PAPVC A CT-angiogram of the pulmonary arteries, coronal plan, revealing the incidental finding of a left partial anomalous pulmonary venous return (red arrows), returning blood to the left brachiocephalic vein (A), which leads to the superior vena cava (B) and the right atrium.

**Figure 3:**
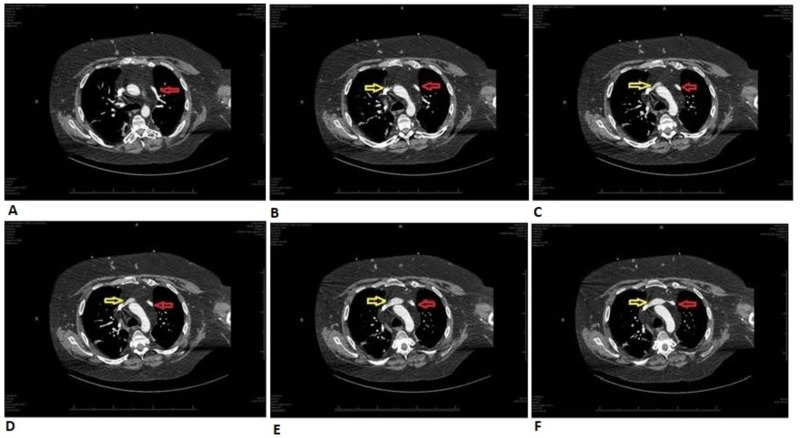
CT-angiogram axial plan showing PAPVC A CT-angiogram of the pulmonary arteries, axial plan, revealing the course (A-F) of the anomalous left superior pulmonary vein (red arrows) until joining the left brachiocephalic vein (yellow arrows).

**Figure 4:**
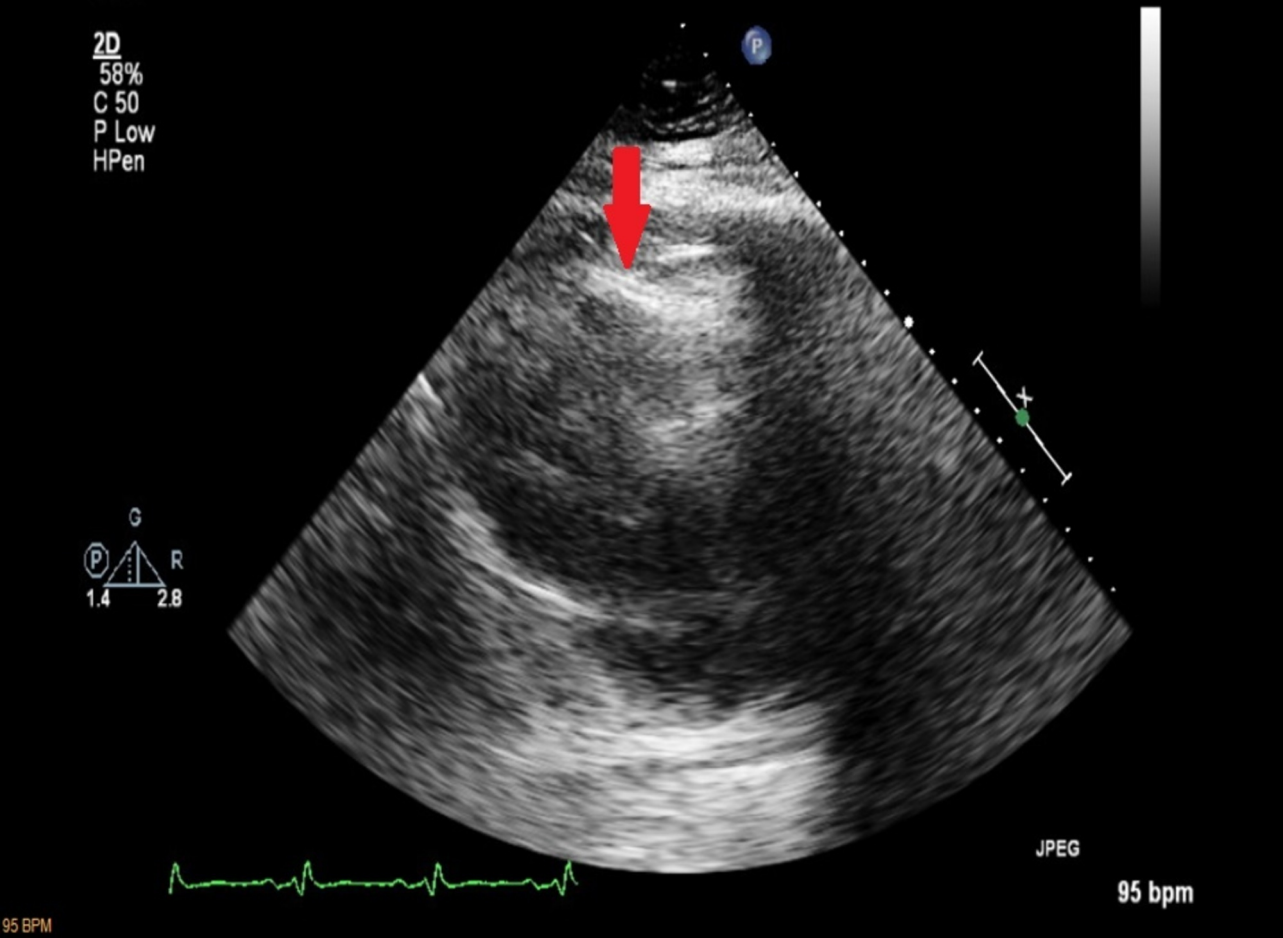
Echocardiogram Apical four chamber echocardiogram view showing mild right ventricular dilatation (red arrow).

A 32-year-old male on methadone for chronic back pain control presented for an outpatient follow-up appointment where he received an EKG for QTc monitoring, revealing an incidental new-onset atrial bigeminy (Figure 1). He was sent to the emergency department for further evaluation. Telemetry and repeat EKG showed resolution of arrhythmia. Given the patient’s complaints of slowly worsening dyspnea requiring supplemental oxygen over a few-month period and new-onset arrhythmia, a CT-angiogram (CTA) of pulmonary arteries was performed. While pulmonary embolism was ruled-out, CTA revealed a PAPVC involving the pulmonary vein in the left upper lobe and lingula, returning blood to the right atrium through the left brachiocephalic vein (Figure 2, Figure 3). An echocardiogram ruled-out ASD but revealed a mildly dilated right ventricle (Figure 4), mild tricuspid valve regurgitation and normal left ventricular function. Notably, the patient had morbid obesity, obstructive sleep apnea, and acute lymphocytic leukemia with bone marrow transplant and graft-versus-host disease causing severe restrictive lung disease (total lung capacity: 42%; FEV1: 39%; FVC: 37%; and DLCO: 41% of predicted values). By that time, the patient was on two liters of oxygen supplementation around the clock at home and he had a functional capacity of four metabolic equivalents (METS). In the setting of the patient’s progressively worsening severe dyspnea requiring further evaluation of the shunt, a right and left heart catheterization was performed. It revealed pulmonary hypertension with pulmonary artery systolic pressure of 52 mmHg, pulmonary artery diastolic pressure of 43 mmHg, mean pulmonary artery pressure of 40 mmHg pulmonary vascular resistance of 1.7 Wood Units, pulmonary capillary wedge pressure of 25 mmHg and cardiac output (Fick) of 7.47 L/min. It also revealed a shunt-index, pulmonary to systemic flow ratio (Qp/Qs), of 1.22:1. Despite his severe symptoms and echocardiogram findings, a multi-disciplinary meeting concluded that the patient would not benefit from surgical intervention. Nine months later, our patient didn’t show signs or symptoms of clinical deterioration. He had no increased requirements for oxygen supplementation and his functional capacity remained stable at four METS.

 

## Discussion

We report an uncommon case of a severely symptomatic young adult male with an incidentally discovered isolated left PAPVC in the setting of underlying severe pulmonary hypertension secondary to multifactorial, severe restrictive lung disease. PAPVC is a rare cardiac anomaly that occurs when one or more of the pulmonary veins drain into a systemic vein. Embryologically, the most accepted theory is that pulmonary bed venous blood initially drains into the splanchnic plexus which is connected to cardinal and umbilicovitelline veins. Later on, regression of primitive connections with systemic veins takes place and splanchnic plexus connects to common pulmonary vein budding from the wall of the left atrium. Failure of this connection results in PAPVC [2]. Classically, PAPVC is associated with ASD in 80%-90% of cases. Isolated PAPVC with an intact atrial septum is an extremely rare entity. However, what makes our case remarkable is the clinical judgment behind the decision to not surgically intervene despite the presence of severe symptoms and the uncertainty about the possible added surgical benefits by the time of decision making. Although theoretically the degree of left-to-right shunting is associated with the severity of symptomatology, pulmonary vascular disease was still found to occur in cases of one PAPVC [3]. Currently, there is no consensus on when to surgically intervene in PAPVC cases [1]. ElBardissi et al. suggested that all patients who have PAPVCs with evidence of right ventricular dilation, mild-to-moderate tricuspid regurgitation, or early stages of pulmonary vascular disease should undergo surgical repair to prevent progression of right ventricular failure and irreversible pulmonary vascular disease [6]. Most centers and literature don’t support intervention for Qp:Qs < 2:1 and isolated PAPVC [10].

In our case, our patient had mild right ventricular dilatation, mild tricuspid regurge and a relatively small shunt-index (Qp:Qs < 2:1) out of proportion to his underlying moderate pulmonary hypertension. The PAPVC was considered to be an insignificant contributor to his symptoms and his pulmonary hypertension was deemed to be multifactorial making him a poor surgical candidate. Furthermore, single anomalous veins are not typically hemodynamically significant in the short-term unless the hemodynamic effect is augmented by concomitant left-to-right shunts like ASDs [5]. On a nine-month follow-up appointment, our patient did not show signs or symptoms of clinical deterioration as expected. His oxygen supplementation requirements remained the same and he had a functional capacity of four METS.

## Conclusions

The presence of a PAPVC in a symptomatic patient should not predetermine surgical intervention. Decision-making in such cases should take into account the nature of the PAPVC, shunt-index severity, its contribution to the overall clinical picture, underlying comorbid conditions, patient’s life-expectancy, and complications associated with PAPVC repair. If a decision is made to defer surgical intervention, strict follow-up and repeat re-evaluations for possible risk re-stratification and surgery reconsideration are warranted.
